# Finite element simulation of Soret-Dufour and conjugate heating effects on mixed convective heat absorbing hydromagnetic Casson fluid flow with suction/blowing from flat semi-infinite vertical porous plate

**DOI:** 10.1016/j.heliyon.2024.e24150

**Published:** 2024-01-08

**Authors:** B. Prabhakar Reddy, Alijen Felician, P.M. Matao

**Affiliations:** Department of Mathematics and Statistics, The University of Dodoma, P. O. Box 338, Dodoma, Tanzania

**Keywords:** Hydromagnetic, Suction/blowing, Porous medium, Chemical reaction, Conjugate heating, Soret-Dufour effect

## Abstract

The finite element simulations of the Soret-Dufour and angled magnetic field effects on conjugate heat and mass transportation of unsteady heat absorbing hydromagnetic Casson flow across a semi-infinite flat oscillatory plate engrafted in a porous medium with suction/blowing, radiation and chemical reaction is performed. The dimensionless coupled flow guiding nonlinear PDEs of the physical structure is handled numerically by the dynamic Galerkin finite element scheme. The demeanor of the velocity, concentration and the temperature profiles due to the alterations in the regulating flow parameters are examined graphically whereas the wall-friction, mass and heat transfer rates explicated by utilizing the tabular data. The research discovered that radiation; conjugate heat transfer and diffusion thermo effects heighten the temperature and velocity distributions whereas heat absorption has a reverse effect. Likewise, conjugate mass transfer and thermo-diffusion effects intensify the concentration and velocity distributions whereas the chemical reaction display overturns aspect. Increased radiation absorption, inclined magnetic field and porosity parameter stimulate fluid velocity whereas the Casson and magnetic parameters exhibit the converse impact. In the instance of suction, the profiles of concentration, velocity and temperature displayed a downturn nature but for the case of blowing, it was noticed a reversal trend. Further, a comparative analysis between the current findings and existing research works in the literature demonstrates our results are exact and accurate.

## 1 Introduction

The problem of magneto-hydrodynamic (MHD) Casson flow provides the groundwork for understanding the complexion of electrically guiding non-Newtonian fluids in the incidence of magnetic fields. This particular type of fluid motion is encountered in various industry and engineering contexts, such as chemical processing, biomedical engineering, and polymer manufacturing. The investigation of MHD Casson flow dominance the significant importance in effective industrial processes and devising innovative technologies. Nonetheless, the complexity of the fluid dynamics involved makes it a formidable task, necessitating the use of advanced mathematical and computational methods for modeling and analysis. The examination of MHD Casson liquid migration has earned global attention due to its influential applications in the study of magnetic drug targeting, where magnetic fields are employed to guide drugs to specific areas of the body, and in the designing of magnetic pumps, which can move fluids without the need of physical contact between the pump and the fluid, relying solely on magnetic fields. Keeping in mind all these utilizations, Krishna et al. [1] studied reactive Casson fluid flow through a stretched porous sheet. Kataria and Patel [2] analyzed the heat and mass characteristics of MHD Casson fluid in a porous channel via an oscillating upward plate. Makinde et al. [3] focused on energy transfer on a 2-D Casson fluid passage due to the uppermost horizontal plate surface of a thermally stratified paraboloid revolution. An extensive research investigation was conducted on MHD Casson fluid transfer under diverse contexts by Refs. [[4], [5], [6], [7], [8], [9]]. Subsequently, Asogwa and Ibe [10] developed the MATLAB bvp4c solver to explore the domination of heat and mass transfer by the flow of MHD Casson fluid towards a permeable stretching plane surface. Several other research studies [[11], [12], [13], [14]] have specifically focused on the effects of radiation, heat-source along with other physical parameters on the flow of MHD Casson fluid via the different porous surfaces. Very recently, Investigations [15,16] discussed the behavior of Casson nanofluid under different circumstances through exponentially stretching and bidirectional stretching surfaces.

The dominance of thermal radiation and chemical reactions in flow with MHD can lead to complex dynamics as these two phenomena interact with each other. The behavior exhibited in such situations is highly dependent on specific conditions and parameters of the flow, including the strength of the magnetic force, radiation intensity, and characteristics of the chemical reactions involved. However, the flow of MHD radiative and reactive liquid extends to numerous influential appliances in engineering, sciences, and industries, such as heat exchangers, irrigation pumps, cooling nuclear reactors, and food processing. Due to the specified significant applications, researchers [[17], [18], [19], [20], [21], [22], [23], [24], [25], [26]] elaborately analyzed the repercussions of chemical reaction and thermal radiation on MHD fluid momentum by considering various fluids in a porous material under distinctive flow geometrics. Further, Suresh et al. [27] discussed radiative and reactive MHD Casson fluid heat and mass transport characteristics via porous substance flowing along an exponentially stretching sheet surface. Bejawada et al. [28] marked thermal radiation consequence in reactive MHD Casson liquid streaming over an inclined non-linear surface.

When a fluid flows over a plate surface, frequently results in a temperature disparity between the fluid and the plate for multiple reasons, including heat exchange with the surroundings, frictional heating, or chemical reactions. These kinds of temperature differences lead to heat transfer along with the fluid and the plate, influencing both the flow and heat conduction properties of the structure. This problem is commonly referred as the conjugate heating effect. This phenomenon finds momentous applications in various scientific and engineering fields, including mechanics, aerospace, nuclear engineering, biology, and meteorology. Many noteworthy investigations [[29], [30], [31], [32], [33], [34], [35], [36]] scrutinized the aftermath of Newtonian heating (NH) on heat and mass transfer of radiating MHD chemically reacting Casson fluid flowing through different porous materials. Mahato and Das [37] purposely examined the repercussions of conjugate heating aspect on unsteady Casson heat captivating and radiating fluid flowing over a semi-infinite flat porous surface by considering chemical reaction, suction/blowing, inclined magnetic field. Afterwards, several studies [[38], [39], [40], [41]] focused on an exploration of Newtonian heating impact on magneto-hydrodynamic Casson flow by including various surface conditions.

The relationship between fluxes and driving materials grows more complicated while heat and mass transfer become obvious simultaneously via MHD Casson fluid. The diffusion-thermo (Dufour) effect emerges from the concentration gradient and produces an energy flux in addition to temperature gradients. Equivalently, the thermal-diffusion (Soret) effect expedites mass flux in response to temperature gradients. Although, the Soret-Dufour effects are typically neglected in many heat and mass transfer examinations owing to their smaller magnitude related to Fourier and Fick’s laws. However, Soret-Dufour effects can play a decisive role in the fields such as petrology, chemical engineering, geosciences, geothermal energy, and nuclear actor research. For instance, the Soret effect is practiced in isotope severance between the mixtures with gases of different molecular weights. Several researchers [[42], [43], [44], [45], [46]] checked the Soret-Dufour effects with other embedded parameters on the Casson unsteady fluid flowing via the vertical porous plate. Subsequently, Bejawada and Yanala [47] employed the finite element technique to explore the impact of the Soret-Dufour effect on unsteady MHD flow with heat and mass transportation from a quickened inclined vertical plate. The investigators [48,49] have purposely investigated Dufour-Soret’s influence on the mixed convection of magneto-hydrodynamic radiating and reacting Casson fluid flowing through different media. Reddy et al. [50] investigated the sway of multiple slips, Dufour-Soret and suction/blowing effects on MHD unsteady flow throughout a non-isothermal stretched surface. Prabhakar Reddy et al. [51] enforced the finite element numerical procedure to highlight the effects of diffusion-thermal on the mixed convection reacting unsteady MHD Casson fluid in the direction of an oscillating porous plate. Recently, Reddy et al. [52] utilized a numerical technique to prospect the combined persuade of radiation and Soret-Dufour’s parameters on natural convection MHD unsteady flow of Casson fluid via a semi-infinite vertical porous surface.

The above scrupulous literature evaluation exposed that the problem of conjugate heat and mass transmute mixed convective radiation absorption oscillatory hydro-magnetic flow of radiating Casson fluid across a flat semi-infinite surface entrenched in a porous medium with an angled magnetic field, thermo-diffusion, heat absorption, chemical reaction and diffusion-thermo in the existence of suction/blowing, no scientific study is yet conducted. However, this kind of research has significant usages in the chemical industry, printing mechanization, polymer, mechanical filtering, pharmaceutical, textile, food manufacturing, drag-reducing agents, and medical sciences and in the production of dumping accessories, etc. Because of these listed significant applications and the quintessence of mixed convective heat absorbing hydro-magnetic oscillatory Casson fluid with conjugate heating effects, the main goal of the authors in the ongoing study is to interpret the effects of inclined magnetic field, radiation absorption, thermo-diffusion, radiation and diffusion-thermo on mixed convection chemically reacting hydro-magnetic Casson flow of heat absorptive fluid from the flat semi-infinite porous oscillating plate with suction/blowing. This model problem has not been previously examined. Hence, our present study is unique. The popular Galerkin finite element scheme is utilized to simulate the non-dimensional matched nonlinear PDEs of the prospective physical model. This numerical technique coupled through the model which in epitome broadens the past work of Mahato and Das [37] by incorporating the radiation absorption, Soret and Dufour effects including its paramount applications encompass the novelty of the current research.

## 2 Mathematical description

**Fig. 1:**
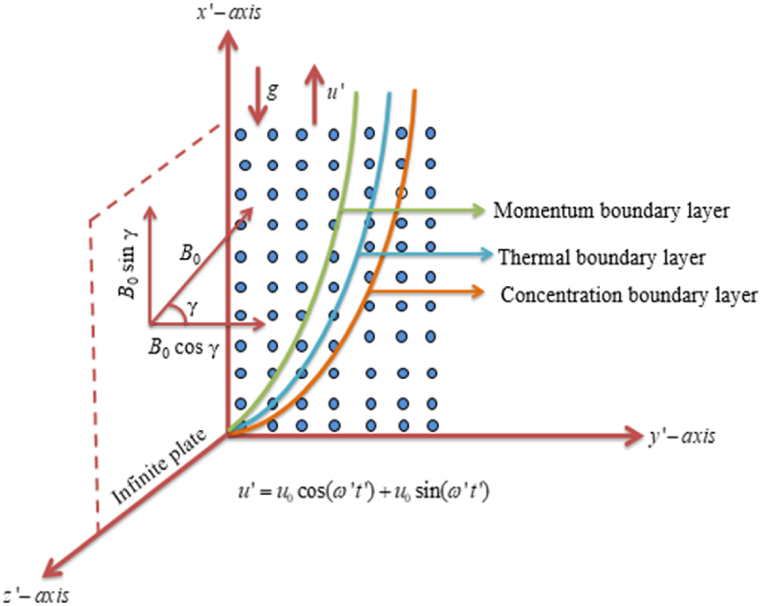
Flow geometry.

Consider a mixed convection 2D hydro-magnetic viscous, radiation absorption incompressible unsteady radiating Casson fluid flow of electrically conducting, chemically reactive species through a flat semi-infinite vertical plate implanted in a porous media with aspects of thermo-diffusion, heat absorption, diffusion-thermo, and suction/blowing. The characteristics of heat and mass transmit are explained by the conjugate heat and mass transfer processes. Let $x^{\prime }-$ axis be aligned vertically with the plate surface, $y^{\prime }-$ axis is perpendicular to it and $z^{\prime }-$ axis normal to plane $x^{\prime }y^{\prime }$ as presented in Fig. 1. At $t^{\prime }=0$ the plate and fluid both are caught up with constant temperature and concentration $T_{\infty }^{\prime }$ and $C_{\infty }^{\prime }\text{,}$ respectively. Subsequently, for $t^{\prime }>0\text{,}$ the plate instigate to oscillate in its own plane with the velocity $u=u_{0}\;\cos\left(\omega 't'\right)+u_{0}\;\sin\left(\omega 't'\right)$ oppositely the gravitational force. The Casson fluid’s constitutive equation can be conveyed following [10,37] as:

(1) $$\tau_{ij}=\left\{ \begin{array}{c} 2\left(\mu_{B}+\frac{p_{y}}{\sqrt{2\pi }}\right)e_{ij}\;\text{when}\;\pi >\pi_{c},\\ 2\left(\mu_{B}+\frac{p_{y}}{\sqrt{2\pi_{c}}}\right)e_{ij}\;\text{when}\;\pi <\pi_{c}. \end{array}\right.$$

In the above Eq. (1), $\pi =e_{ij}e_{ij}$ and $e_{ij}-$ indicates ${\left(i,j\right)}^{th}$ the rate of deformity component, $\pi_{c}-$ indicate the critical charge grounded on the non-Newtonian model, π− indicate product of self deformation rate, and $p_{y}-$ indicate fluid’s yield stress.

The following assumptions are made in the current inspection:

(i) A continuous magnetic field $B=\left(B_{0}\;\cos\;\gamma ,B_{0}\;\sin\;\gamma ,0\right)$ is enforced in $y^{\prime }$-direction with an angle γ with $y^{\prime }>0$.
(ii) The magnetic Reynolds number estimated to be paltry; hence the importance of the magnetic field infused by flow impetus is ignored.
(iii) There is no externally sanctioned electric field, so that the response of polarization is overlooked.
(iv) The fluid flow is originated by the plate impulsive motion in $x^{\prime }-$ direction and due to the pores in the plate surface there is no constant flow in $y^{\prime }-$ direction, hence the velocity vector is given by $q=\left(u^{\prime },-\upsilon_{0},0\right)$.

By the foregoing assumptions, in addition to the usual Boussinesq’s approximation, the set of flow regulating coupled PDEs of physical phenomena with initial and boundary conditions following [37,47] derived as;

(2) $$\frac{\partial u^{\prime }}{\partial t^{\prime }}-\upsilon_{0}\frac{\partial u^{\prime }}{\partial y^{\prime }}=\nu \left(\frac{1}{\beta }+1\right)\frac{\partial^{2}u^{\prime }}{\partial {y^{\prime }}^{2}}-\frac{\sigma B_{0}^{2}\;\cos^{2}\;\gamma }{\rho }u^{\prime }-\nu \left(\frac{1}{\beta }+1\right)\frac{u^{\prime }}{K^{\prime }}+g\beta^{T}\left(T^{\prime }-T_{\infty }^{\prime }\right)+g\beta^{C}\left(C^{\prime }-C_{\infty }^{\prime }\right)\text{,}$$

(3) $$\frac{\partial T^{\prime }}{\partial t^{\prime }}-\upsilon_{0}\frac{\partial T^{\prime }}{\partial y^{\prime }}=\frac{k}{\rho c_{p}}\frac{\partial^{2}T^{\prime }}{\partial {y^{\prime }}^{2}}-\frac{Q_{0}}{\rho c_{p}}\left(T^{\prime }-T_{\infty }^{\prime }\right)+\frac{Q_{1}}{\rho c_{p}}\left(C^{\prime }-C_{\infty }^{\prime }\right)-\frac{1}{\rho c_{p}}\frac{\partial q_{r}}{\partial y^{\prime }}+\frac{D_{m}K_{T}}{c_{p}c_{s}}\left(\frac{\partial^{2}C^{\prime }}{\partial {y^{\prime }}^{2}}\right)\text{,}$$

(4) $$\frac{\partial C^{\prime }}{\partial t^{\prime }}-\upsilon_{0}\frac{\partial C^{\prime }}{\partial y^{\prime }}=D_{m}\frac{\partial^{2}C^{\prime }}{\partial {y^{\prime }}^{2}}-k_{c}^{\prime }\left(C^{\prime }-C_{\infty }^{\prime }\right)+\frac{D_{m}K_{T}}{T_{m}}\left(\frac{\partial^{2}T^{\prime }}{\partial {y^{\prime }}^{2}}\right)\text{.}$$

and

(5) $$\begin{array}{l} u^{\prime }=0,T^{\prime }=T_{\infty }^{\prime },C^{\prime }=C_{\infty }^{\prime }\;\text{for}\;y^{\prime }\geq 0\;\text{and}\;t^{\prime }\leq 0\text{,}\\ u^{\prime }=u_{0}\;\cos\left(\omega 't'\right)+u_{0}\;\sin\left(\omega 't'\right),\frac{\partial T^{\prime }}{\partial y^{\prime }}=-h_{1}T^{\prime },\frac{\partial C^{\prime }}{\partial y^{\prime }}=-h_{2}C^{\prime }\;\text{at}\;y^{\prime }=0\;\text{for}\;t^{\prime }>0\text{,}\\ u^{\prime }\to 0,T^{\prime }\to T_{\infty }^{\prime },C^{\prime }\to C_{\infty }^{\prime }\;\text{as}\;y^{\prime }\to \infty \text{for}\;t^{\prime }>0\text{.} \end{array}\}$$

here, $\nu ,\beta ,T^{\prime },\sigma ,K^{\prime },g,\rho ,\beta^{T},u^{\prime },t^{\prime },\upsilon_{0},T_{\infty }^{\prime },y^{\prime },\beta^{C},C^{\prime },C_{\infty }^{\prime },k,u_{0},c_{p},Q_{0},Q_{1},q_{r},D_{m},K_{T},c_{s},k_{c}^{\prime },T_{m},h_{1},\omega t,h_{2}\text{,}$ and $B_{0}\text{,}$ respectively indicates the kinematic viscosity, Casson parameter, fluid temperature, electrical conductivity, medium permeability, gravitational force, fluid density, thermal extension coefficient, fluid’s velocity, time, fluid viscosity, fluid temperature distantly from the plate, span-wise coordinate, concentration extension coefficient, fluid’s concentration, fluid concentration distantly from the plate, thermal conductivity, amplitude of the plate, specific heat at steady pressure, heat absorption, radiation absorption, radiation heat flux, molecular diffusivity, thermo-diffusion ratio, concentration susceptibility, chemical reaction constant, mean value temperature, heat transfer coefficient, phase angle, mass transfer coefficient and the magnetic field intensity.

With the assumption that fluid’s temperature is linearly related to thermal conductivity, then the radiation flux vector $q_{r}$ in Equation (3) can be written by following Rosseland approximation [53] as:

(6) $$q_{r}=-\;\frac{\;4\sigma_{s}}{3A_{m}}\frac{\partial {T^{'}}^{4}}{\partial y^{'}}$$

here, $A_{m}$ and $\sigma_{s}$ are indicate respectively, the mean absorptive and Stephen Boltzmann constants. We presume that the temperature changes inside the flow allow us to expand ${T^{\prime }}^{4}$ by engaging the Taylor series expansion about $T_{\infty }^{\prime }$, upon overlooking higher order terms (i.e., from 2nd order terms), bring about;

(7) $${T^{\prime }}^{4}≅4{T^{\prime }}_{\infty }^{3}T^{\prime }-3{T^{\prime }}_{\infty }^{4}$$

After using Eqs. (6), (7) into Eq. (3), results

(8) $$\frac{\partial T^{\prime }}{\partial t^{\prime }}-\upsilon_{0}\frac{\partial T^{\prime }}{\partial y^{\prime }}=\frac{k}{\rho c_{p}}\frac{\partial^{2}T^{\prime }}{\partial {y^{\prime }}^{2}}-\frac{Q_{0}}{\rho c_{p}}\left(T^{\prime }-T_{\infty }^{\prime }\right)+\frac{Q_{1}}{\rho c_{p}}\left(C^{\prime }-C_{\infty }^{\prime }\right)+\frac{16\sigma_{s}{T^{\prime }}_{\infty }^{3}}{3\rho c_{p}A_{m}}\left(\frac{\partial^{2}T^{\prime }}{\partial {y^{\prime }}^{2}}\right)+\frac{D_{m}K_{T}}{c_{p}c_{s}}\left(\frac{\partial^{2}C^{\prime }}{\partial {y^{\prime }}^{2}}\right)$$

We define the following dimensionless variables and parameters:

(9) $$\begin{array}{c} \xi =y^{'}\frac{u_{0}}{\nu },u=\frac{u^{'}}{u_{0}},t=t^{'}\frac{u_{0}^{2}}{\nu },\theta =\frac{T^{'}-T_{\infty }^{'}}{T_{\infty }^{'}},S=\frac{\upsilon_{0}}{u_{0}},K_{c}=\frac{k_{c}}{u_{0}^{2}},\gamma_{1}=\frac{h_{1}\nu }{u_{0}}\text{,}\\ M=\frac{\sigma B_{1}^{2}\nu }{\rho u_{0}^{2}},G_{r}=\frac{\rho \beta^{T}\nu T_{\infty }^{'}}{u_{0}^{3}},G_{m}=\frac{\rho \beta^{C}\nu C_{\infty }^{'}}{u_{0}^{3}},P_{r}=\frac{\rho \nu c_{p}}{k},\omega =\omega^{'}\frac{\nu }{u_{0}^{2}}\text{,}\\ K=K^{'}\frac{u_{0}^{2}}{\nu^{2}},S_{c}=\frac{\nu }{D_{m}},\varphi_{1}=\frac{\nu Q_{0}}{\rho c_{p}u_{0}^{2}},\varphi_{2}=\frac{\nu Q_{1}C_{\infty }^{'}}{\rho c_{p}u_{0}^{2}T_{\infty }^{'}},N_{r}=\frac{16\sigma_{s}T_{\infty }^{'}}{3kA_{m}}\text{,}\\ C=\frac{C^{'}-C_{\infty }^{'}}{C_{\infty }^{'}},D_{u}=\frac{K_{T}D_{m}C_{\infty }^{'}}{c_{p}c_{s}\nu T_{\infty }^{'}},S_{r}=\frac{K_{T}D_{m}T_{\infty }^{'}}{T_{m}\nu C_{\infty }^{'}},\gamma_{2}=\frac{h_{2}\nu }{u_{0}}\text{.} \end{array}\}$$

Submitting Eq. (9), into Equations (2), (4), (5), (8) designate into the following dimensionless nonlinear PDEs of the stated flow with initial and boundary specifies:

(10) $$\frac{\partial u}{\partial t}-S\frac{\partial u}{\partial \xi }=\left(\frac{1}{\beta }+1\right)\frac{\partial^{2}u}{\partial \xi^{2}}-\left[M\;\cos^{2}\;\gamma +\left(\frac{1}{\beta }+1\right)\frac{1}{K}\right]u+G_{r}\theta +G_{m}C\text{,}$$

(11) $$\frac{\partial \theta }{\partial t}-S\frac{\partial \theta }{\partial \xi }=\left(\frac{1+N_{r}}{P_{r}}\right)\frac{\partial^{2}\theta }{\partial \xi^{2}}-\varphi_{1}\theta +\varphi_{2}C+D_{u}\left(\frac{\partial^{2}C}{\partial \xi^{2}}\right)\text{,}$$

(12) $$\frac{\partial C}{\partial t}-S\frac{\partial C}{\partial \xi }=\frac{1}{S_{c}}\frac{\partial^{2}C}{\partial \xi^{2}}-K_{c}C+S_{r}\left(\frac{\partial^{2}\theta }{\partial \xi^{2}}\right)\text{.}$$

and

(13) $$\begin{array}{l} u=0,\theta =0,C=0\;\text{for}\;\text{all}\;\xi \geq 0\;\text{and}\;t\leq 0\text{,}\\ u=\cos\left(\omega t\right)+\sin\left(\omega t\right),\frac{\partial \theta }{\partial \xi }=-\gamma_{1}\left(\theta +1\right),\frac{\partial C}{\partial \xi }=-\gamma_{2}\left(C+1\right)\;\text{at}\;\xi =0\;\text{and}\;t>0\text{,}\\ u\to 0,\theta \to 0,C\to 0\;\text{for}\;\xi \to \infty \text{and}\;t>0\text{.} \end{array}\}$$

here, $t,S,N_{r},\beta ,u,M,C,\varphi_{1},K,G_{r},S_{c},\xi ,\theta ,\gamma ,G_{m},P_{r},\varphi_{2},D_{u},K_{c}$ and $S_{r}$ respectively, indicates the dimensionless time, suction/blowing, radiation parameter, Casson parameter, fluid velocity, magnetic parameter, fluid concentration, heat absorption parameter, porosity parameter, thermal Grashof number, Schmidt number, dimensionless space coordinate, fluid temperature, magnetic field inclination angle, mass Grashof number, Prandtl number, radiation absorption parameter, Dufour parameter, chemical reaction parameter, and the Soret parameter.

The engineering depiction of quantities of physical significance in the dimensionless form at the plate defined by

Skin friction $\tau =-\left(\frac{1}{\beta }+1\right){\left(\frac{\partial u}{\partial \xi }\right)}_{\xi =0}$,

Heat transfer rate (Nusselt number) $N_{u}=-\left(1+N_{r}\right){\left(\frac{\partial \theta }{\partial \xi }\right)}_{\xi =0}$,

Mass transfer rate (Sherwood number) $S_{h}=-{\left(\frac{\partial C}{\partial \xi }\right)}_{\xi =0}$.

## 3 Numerical technique

This article section discusses the development and implementation of the Galerkin finite element scheme on a non-linear system of three linked PDEs (10)–(12), conjointly with initial and boundary conditions Eq. (13) that describes the proposed physical phenomenon. The numerical method presented here has a broad range of applications and can be effectively implemented to solve various complex problems in the fields of biomechanics, metallurgical transport exercises, mixed convection micro-polar heat transfer problems, third-grade visco-elastic hydrodynamics, magneto-biofluid dynamics, etc. Implementation of the Galerkin finite element scheme on Eqs. (10), (11), (12) over the two-node linear element $\left(e\right),\left(\xi_{j}\leq \xi \leq \xi_{k}\right)$ leads to the following equations:

(14) $$\int_{\xi_{j}}^{\xi_{k}}\psi^{{\left(e\right)}^{T}}\left[\left(1+\frac{1}{\beta }\right)\frac{\partial^{2}u^{\left(e\right)}}{\partial \xi^{2}}-\frac{\partial u^{\left(e\right)}}{\partial t}+S\frac{\partial u^{\left(e\right)}}{\partial \xi }-\Delta_{1}u^{\left(e\right)}+\Delta_{2}\right]d\xi =0\text{,}$$

(15) $$\int_{\xi_{j}}^{\xi_{k}}\psi^{{\left(e\right)}^{T}}\left[\left(\frac{1+N_{r}}{P_{r}}\right)\frac{\partial^{2}\theta^{\left(e\right)}}{\partial \xi^{2}}-\frac{\partial \theta^{\left(e\right)}}{\partial t}+S\frac{\partial \theta^{\left(e\right)}}{\partial \xi }-\varphi_{1}\theta^{\left(e\right)}+\varphi_{2}C+D_{u}\left(\frac{\partial^{2}C}{\partial \xi^{2}}\right)\right]d\xi =0\text{,}$$

(16) $$\int_{\xi_{j}}^{\xi_{k}}\psi^{{\left(e\right)}^{T}}\left[\left(\frac{1}{S_{c}}\right)\frac{\partial^{2}C^{\left(e\right)}}{\partial \xi^{2}}-\frac{\partial C^{\left(e\right)}}{\partial t}+S\frac{\partial C^{\left(e\right)}}{\partial \xi }-K_{c}C^{\left(e\right)}+S_{r}\left(\frac{\partial^{2}\theta }{\partial \xi^{2}}\right)\right]d\xi =0\text{,}$$

where $\Delta_{1}=M\;\cos^{2}\;\gamma +\left(1+\frac{1}{\beta }\right)\left(\frac{1}{K}\right),\Delta_{2}=G_{r}\theta +G_{m}C\text{,}$ and $\psi^{\left(e\right)}=\left[\begin{array}{cc} \psi_{j} & \psi_{k} \end{array}\right]$.

In order to lower the order of integration and nonlinearity in above equations; after applying the integration process in Eqs. (14), (15), (16), we obtain the following equations;

(17) $$\int_{\xi_{j}}^{\xi_{k}}\left[\left(1+\frac{1}{\beta }\right)\frac{\partial \psi^{{\left(e\right)}^{T}}}{\partial \xi }\frac{\partial u^{\left(e\right)}}{\partial \xi }+\psi^{{\left(e\right)}^{T}}\left(\frac{\partial u^{\left(e\right)}}{\partial t}-S\frac{\partial u^{\left(e\right)}}{\partial \xi }+\Delta_{1}u^{\left(e\right)}-\Delta_{2}\right)\right]d\xi -{\left[\left(\psi^{{\left(e\right)}^{T}}\right)\frac{\partial u^{\left(e\right)}}{\partial \xi }\right]}_{\xi_{j}}^{\xi_{k}}=0\text{,}$$

(18) $$\int_{\xi_{j}}^{\xi_{k}}\left[\left(\frac{1+N_{r}}{P_{r}}\right)\frac{\partial \psi^{{\left(e\right)}^{T}}}{\partial \xi }\frac{\partial \theta^{\left(e\right)}}{\partial \xi }+\psi^{{\left(e\right)}^{T}}\left(\frac{\partial \theta^{\left(e\right)}}{\partial t}-S\frac{\partial \theta^{\left(e\right)}}{\partial \eta }+\varphi_{1}\theta^{\left(e\right)}-\varphi_{2}C-D_{u}\left(\frac{\partial^{2}C}{\partial \xi^{2}}\right)\right)\right]d\xi -{\left[\left(\psi^{{\left(e\right)}^{T}}\right)\frac{\partial \theta^{\left(e\right)}}{\partial \xi }\right]}_{\xi_{j}}^{\xi_{k}}=0\text{,}$$

(19) $$\int_{\xi_{j}}^{\xi_{k}}\left[\left(\frac{1}{S_{c}}\right)\frac{\partial \psi^{{\left(e\right)}^{T}}}{\partial \xi }\frac{\partial C^{\left(e\right)}}{\partial \xi }+\psi^{{\left(e\right)}^{T}}\left(\frac{\partial C^{\left(e\right)}}{\partial t}-S\frac{\partial C^{\left(e\right)}}{\partial \xi }+K_{c}C^{\left(e\right)}-S_{r}\left(\frac{\partial^{2}\theta }{\partial \xi^{2}}\right)\right)\right]d\xi -{\left[\left(\psi^{{\left(e\right)}^{T}}\right)\frac{\partial C^{\left(e\right)}}{\partial \xi }\right]}_{\xi_{j}}^{\xi_{k}}=0\text{.}$$

We use finite element approximation to formulate the finite element model from Eqs. (17), (18), (19) over the typical $e^{th}$ element $\left(e\right),\left(\xi_{j}\leq \xi \leq \xi_{k}\right)$ is of the form;

(20) $$u^{\left(e\right)}=\psi_{j}u_{j}+\psi_{k}u_{k}\text{,}$$

(21) $$\theta^{\left(e\right)}=\psi_{j}\theta_{j}+\psi_{k}\theta_{k}\text{,}$$

(22) $$C^{\left(e\right)}=\psi_{j}C_{j}+\psi_{k}C_{k}$$

where $u_{j},u_{k},\theta_{j},\theta_{k},C_{j}$ and ${C_{k}}^{\prime }s$ indicates, respectively the velocity, temperature and the concentration components at $j^{th}$ and $k^{th}$ nodes of the $e^{th}$ element $\left(\xi_{j}\leq \xi \leq \xi_{k}\right),\psi_{j}$ and $\psi_{k}$ are the shape functions over the $e^{th}$ element $\left(\xi_{j}\leq \xi \leq \xi_{k}\right)\text{,}$ described as;

(23) $$\psi_{j}=\frac{\xi_{k}-\xi }{\xi_{k}-\xi_{j}}\;\text{and}\;\psi_{k}=\frac{\xi -\xi_{j}}{\xi_{k}-\xi_{j}}$$

Substituting Eqs. (20), (21), (22) into Eqs. (17), (18), (19) with the help of Eq. (23) and use of the boundary conditions (13), after gathering element equations for all elements by inter connectivity rule, results in the following tri-diagonal form of matrix system;

(24) $$\begin{array}{l} Pu=P^{\prime }\\ Q\theta =Q^{\prime }\\ RC=R^{\prime } \end{array}$$

where P,Q and R specify matrices of order n and u,θ,C,P'Q' and $R^{\prime }$ represents column matrices having n components. The above matrix system of Eq. (24) can be simplified successfully by the Gauss-Seidel iteration scheme. Our main intention is to get the converged solution. For this, care has been taken in selecting mesh dimensions in space ξ− direction and time t− direction. Keeping the mesh length fixed at 0.001 in time t− direction, the developed finite element code was run with distinct mesh lengths 0.01,0.02 and 0.05 in space ξ− direction. In this experiment, we do not find any convincing changed values in u, θ and C. This exercise authenticates the solutions are mesh independent. Furthermore, the solution is expected to be converges at any time; the comparative deviation amidst two successive iterations arrives at a specific value, i.e., the iterative operation is terminated when the condition $\left|f_{i}^{m+1}-f_{i}^{m}\right|<10^{-6}$ is satisfied, where f=u,θ,C and m indicates the iterative step. This condition preserves the high accuracy in solving the multi-physical coupled boundary layer equations.

## 4 Validation

Table 1, Table 2 revealed the comparison of our results captured by the Galerkin finite element scheme by considering skin friction τ in the limiting case and Nusselt number $N_{u}$ with available results from the literature. We clearly point out in the limiting case from Table 1 that when $G_{m}=0,\beta \to \infty ,D_{u}=0,M=0,\varphi_{2}=0,S=0\text{,}$ K→∞ and γ=0, the numerical values of τ computed by us is precisely matched with the results of Das et al. [29] and Mahato and Das [37] computed by the Laplace Transform Technique. Also, it is much apparent from Table 2 that the numerical values of $N_{u}$ computed by us when $D_{u}=0$ and $\varphi_{2}=0$ are exalted matching. Therefore, our current results are exactitude and accurate.

**Table 1:** In the limiting case, comparison of the skin-friction τ.

$P_{r}$	$G_{r}$	$\gamma_{1}$	wt	t	Das et al. [29]	Mahato and Das [37]	FEM results
0.35	5	1	0	0.01	5.5818	5.5812	5.581810
0.50	5	1	0	0.01	5.5956	5.5956	5.595609
0.35	10	1	0	0.01	5.5218	5.5205	5.521825
0.35	5	2	0	0.01	5.5027	5.5011	5.502510
0.35	5	1	π/2	0.01	4.4819	4.4819	4.481989
0.35	5	1	0	0.02	3.8620	3.8606	3.862071

**Table 2:** Comparison of the Nusselt number $N_{u}$.

$P_{r}$	$N_{r}$	$\varphi_{1}$	$\gamma_{1}$	t	Mahato and Das [37]		FEM results	
					$N_{u}$ (Suction)	$N_{u}$ (Blowing)	$N_{u}$ (Suction)	$N_{u}$ (Blowing)
0.71	3	1	0.5	0.7	0.72883814	0.63247729	0.72883814	0.63247729
1.00	3	1	0.5	0.7	0.84550858	0.69516014	0.84550858	0.69516014
0.71	5	1	0.5	0.7	0.62712616	0.57424226	0.62712616	0.57424226
0.71	3	3	0.5	0.7	0.73544219	0.63686326	0.73544219	0.63686326
0.71	3	1	0.7	0.7	0.82210603	0.75708048	0.82210603	0.75708048
0.71	3	1	0.5	0.5	0.78847861	0.69038286	0.78847861	0.69038286

## 5 Results analysis

In this section, we have provided the graphical and tabular representations to demonstrate the features of fluid temperature, concentration, velocity profiles and skin friction, mass and heat transfer rates for the suction and blowing cases due to the diversifications of the flow predominant parameters acting as the magnetic parameter M, energy absorption parameter $\varphi_{1}\text{,}$ Dufour parameter $D_{u}\text{,}$ conjugate heat transfer parameter $\gamma_{1}\text{,}$ radiation absorption parameter $\varphi_{2}\text{,}$ magnetic field inclination angle γ, chemical reaction parameter $K_{c}\text{,}$ porosity parameter K, conjugate mass transfer parameter $\gamma_{2}\text{,}$ Soret parameter $S_{r}\text{,}$ Casson parameter β, phase angle ωt, suction/blowing parameter S, and radiation parameter $N_{r}\text{.}$ Numerical computations were executed using definite constant values of the pertinent parameters, which apply all graphs and tables except for stated otherwise; $N_{r}=2,M=2,D_{u}=0.5,\varphi_{1}=3,K=0.5,\gamma_{1}=0.5,S_{r}=2,\beta =0.5,P_{r}=0.71,K_{c}=2,\varphi_{2}=1,\gamma_{2}=0.5,S_{c}=0.6,\omega t=\pi /3$ and γ=π/6.

**Fig. 2:**
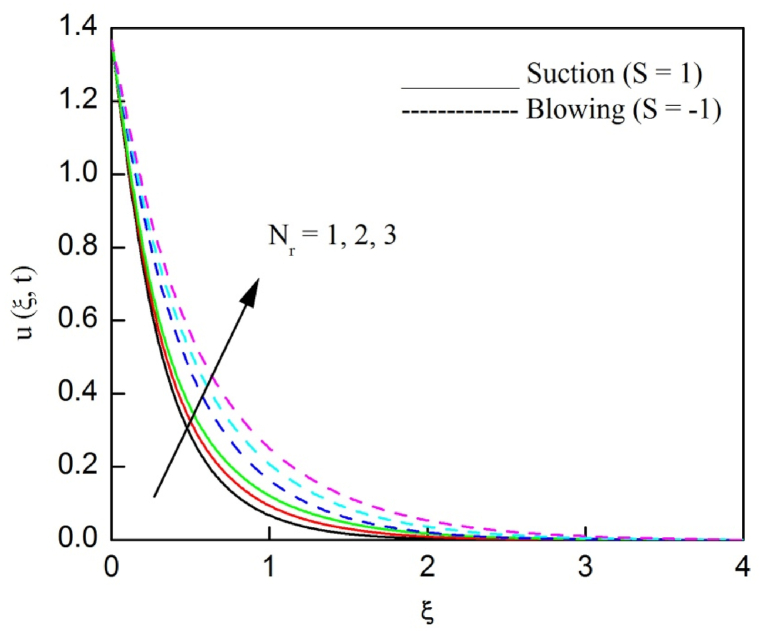
The consequence of $N_{r}$ on velocity distribution vs ξ.

**Fig. 3:**
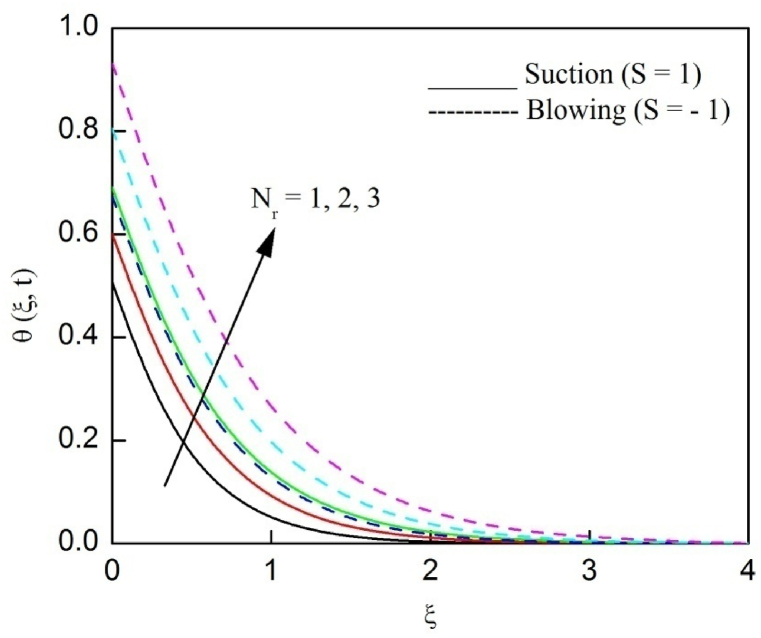
The consequence of $N_{r}$ on temperature distribution vs ξ.

**Fig. 4:**
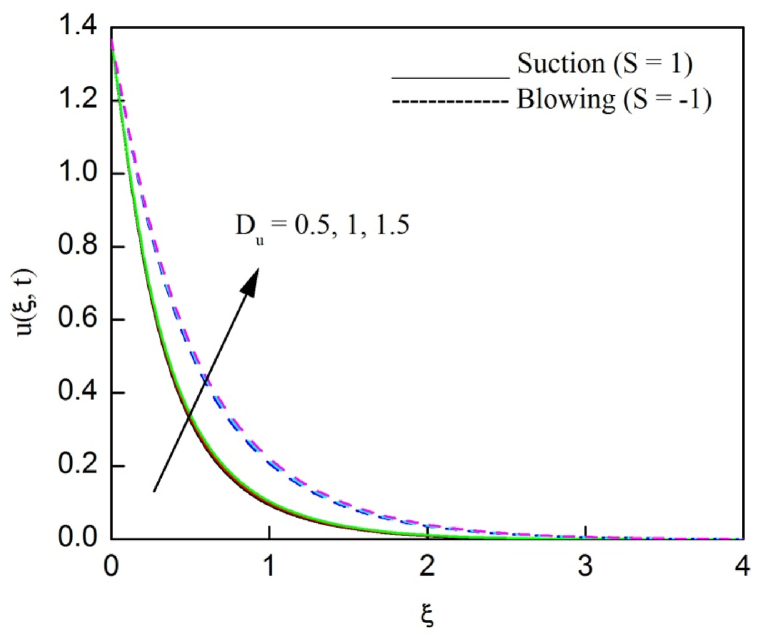
The consequence of $D_{u}$ on velocity distribution vs ξ.

**Fig. 5:**
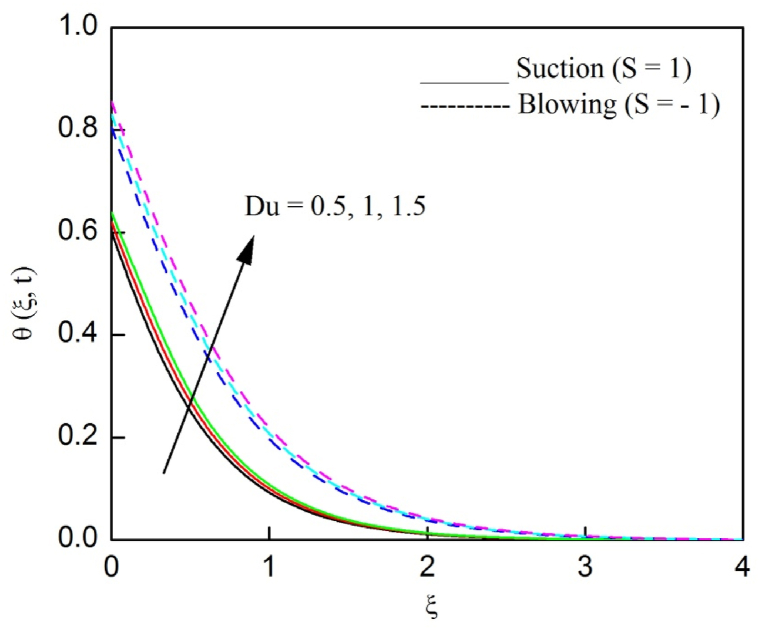
The consequence of $D_{u}$ on temperature distribution vs ξ.

**Fig. 6:**
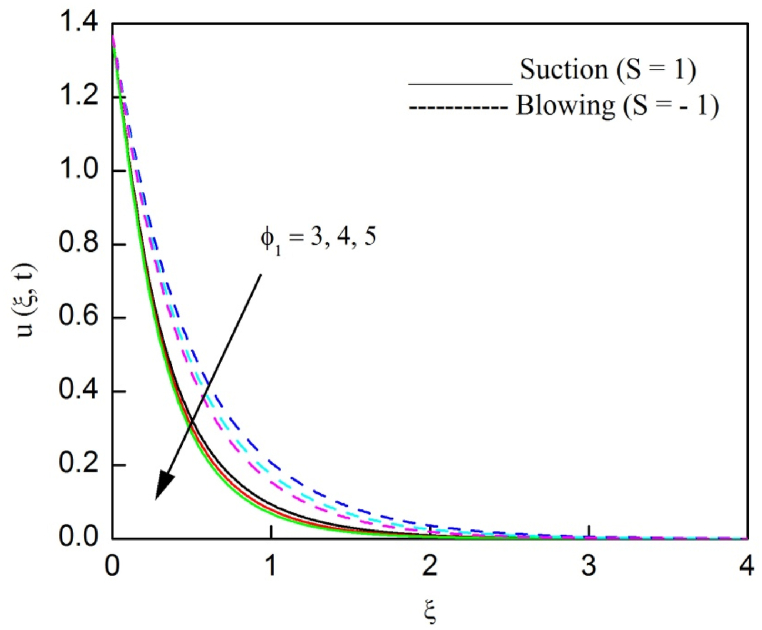
The consequence of $\varphi_{1}$ on velocity distribution vs ξ.

**Fig. 7:**
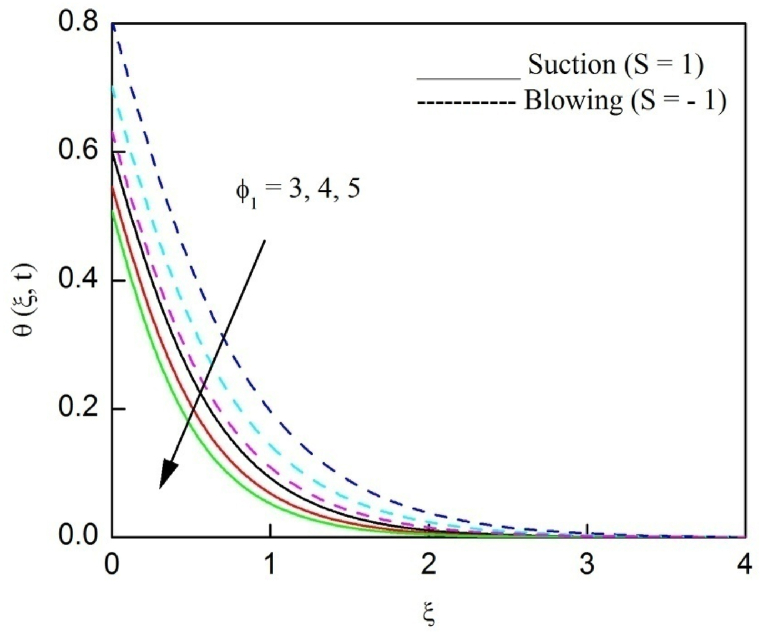
The consequence of $\varphi_{1}$ on temperature distribution vs ξ.

**Fig. 8:**
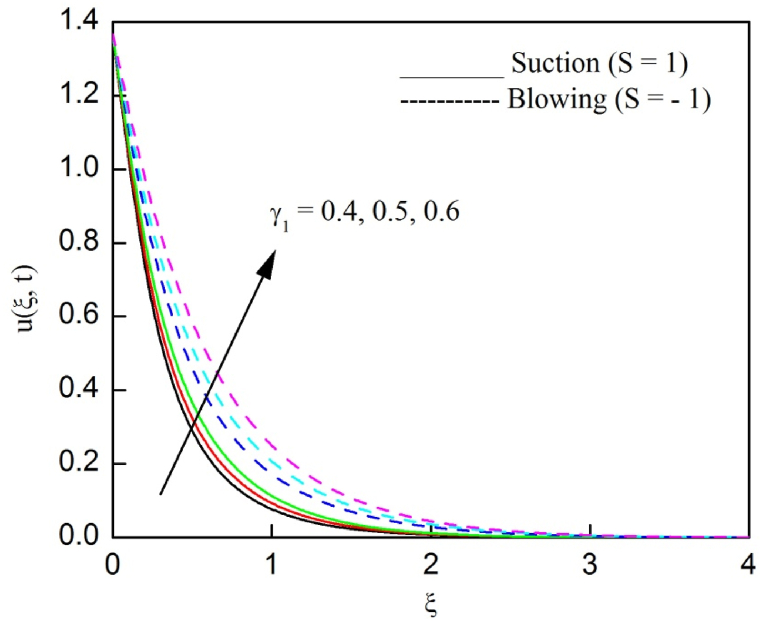
The consequence of $\gamma_{1}$ on velocity distribution vs ξ.

**Fig. 9:**
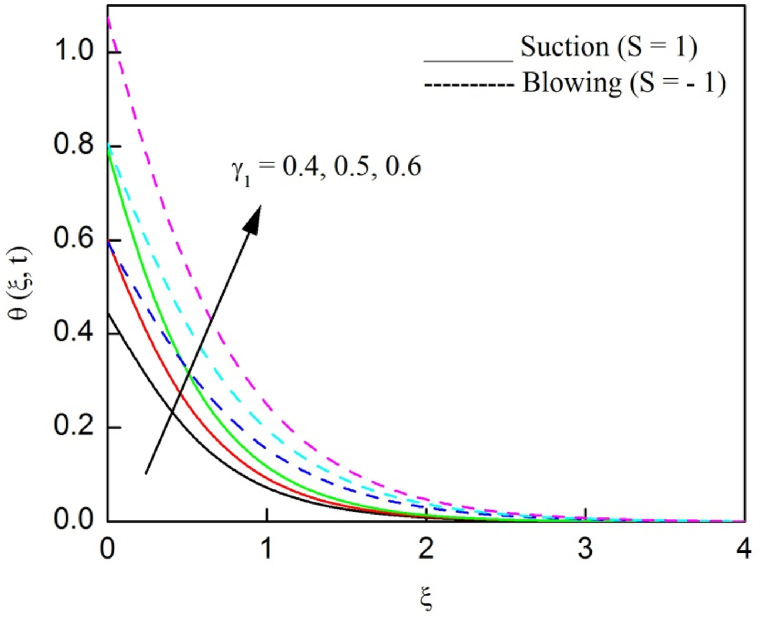
The consequence of $\gamma_{1}$ on temperature distribution vs ξ.

**Fig. 10:**
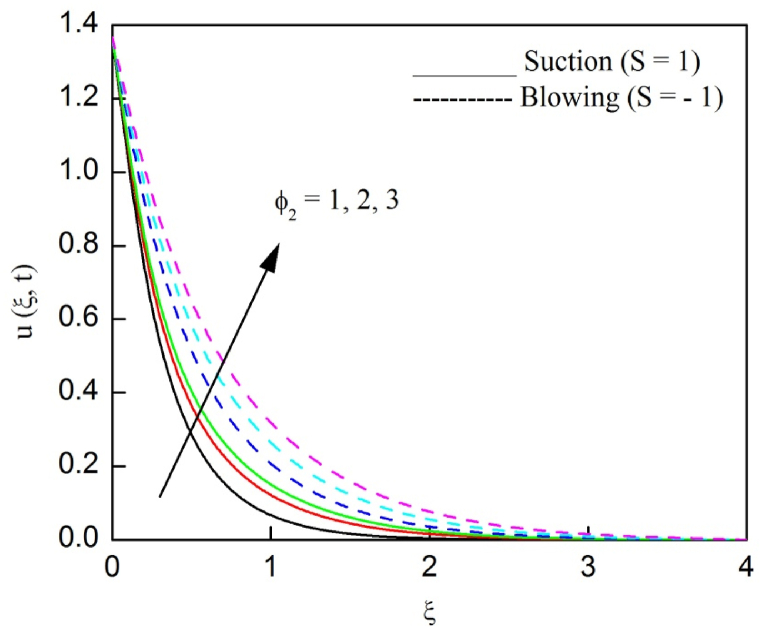
The consequence $\varphi_{2}$ on velocity distribution vs ξ.

**Fig. 11:**
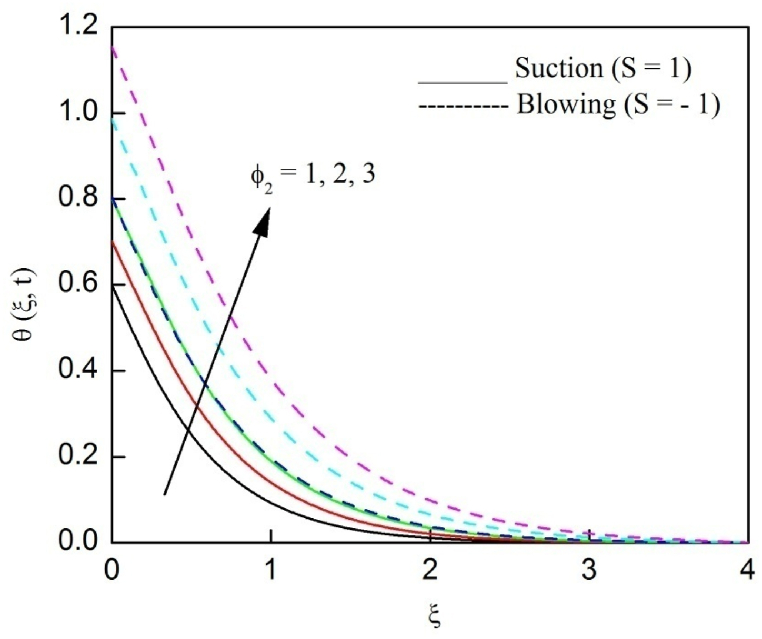
The consequence of $\varphi_{2}$ on temperature distribution vs ξ.

**Fig. 12:**
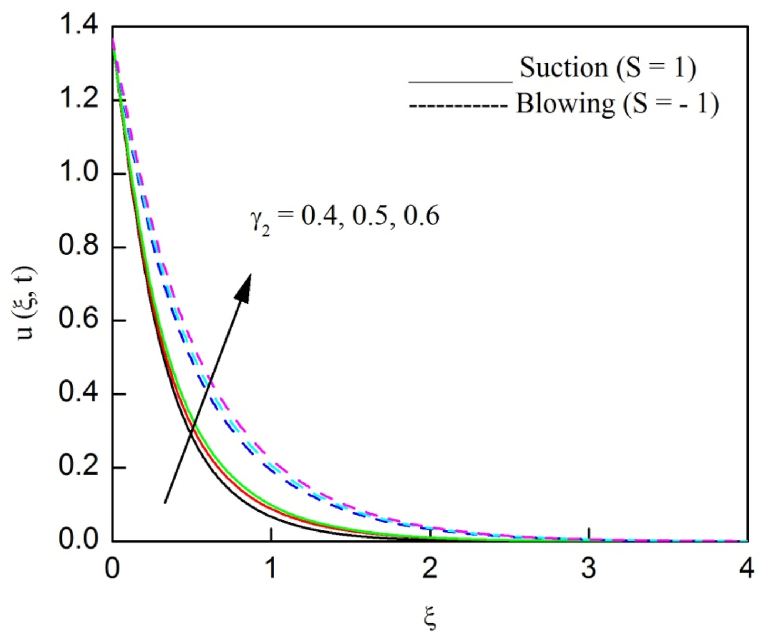
The consequence of $\gamma_{2}$ on velocity distribution vs ξ.

**Fig. 13:**
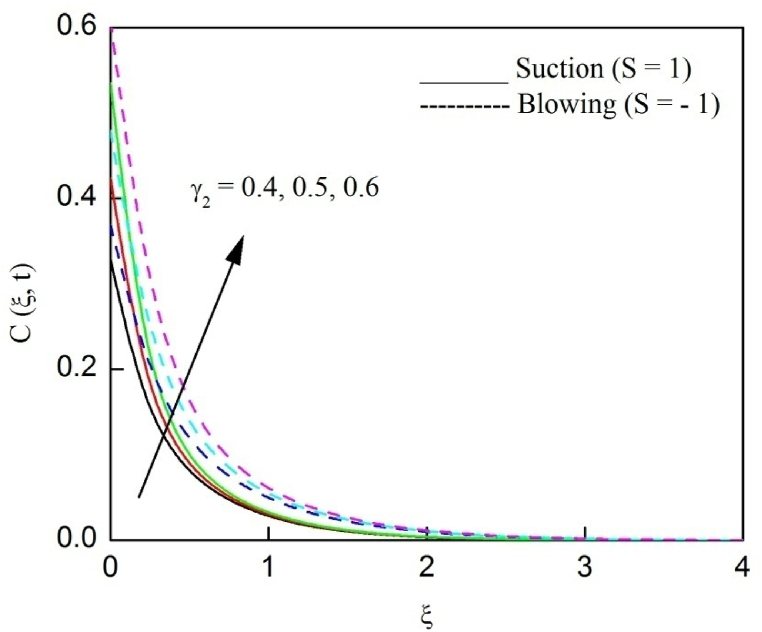
The consequence of $\gamma_{2}$ on concentration distribution vs ξ.

**Fig. 14:**
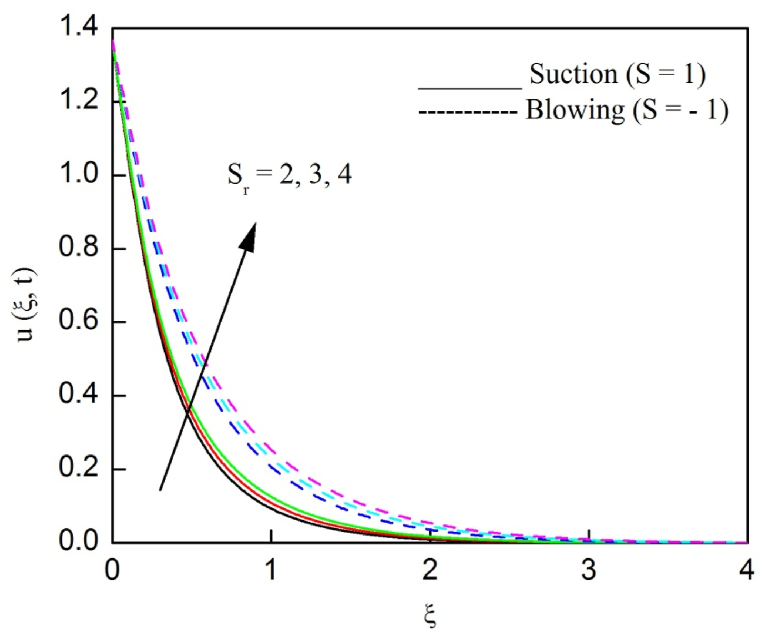
The consequence of $S_{r}$ on the velocity distribution vs ξ.

**Fig. 15:**
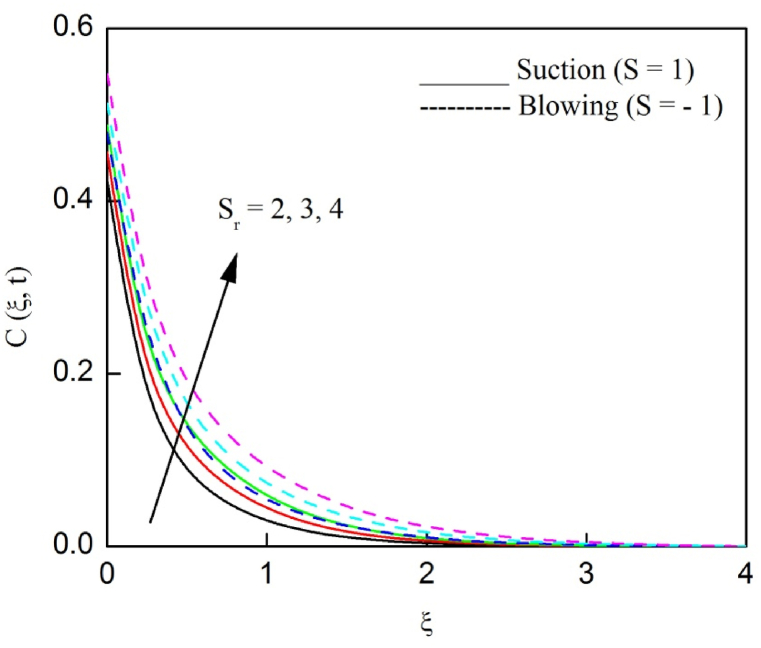
The consequence of $S_{r}$ on concentration distribution vs ξ.

**Fig. 16:**
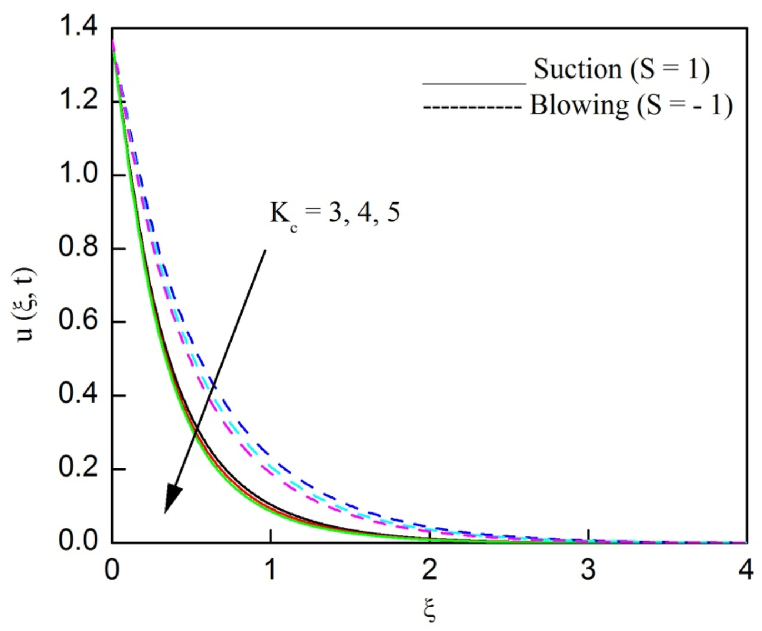
The consequence of $K_{c}$ on velocity distribution vs ξ.

**Fig. 17:**
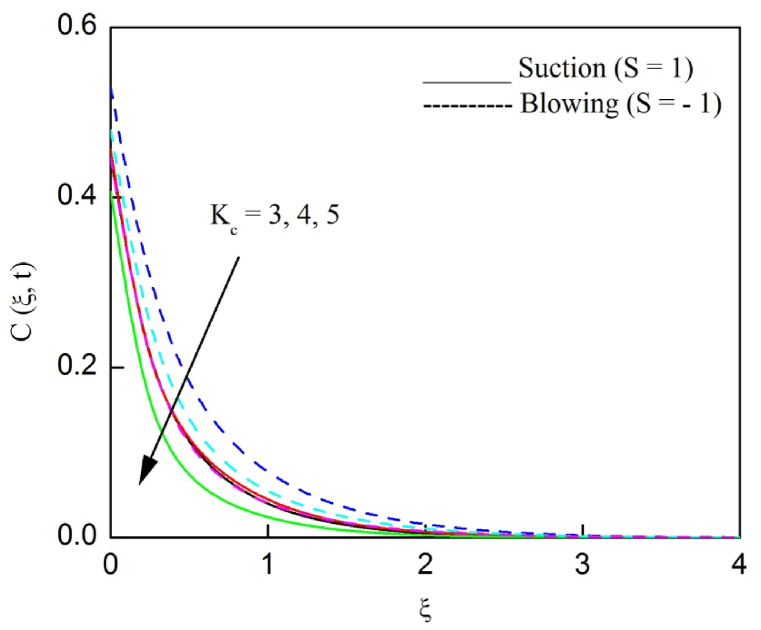
The consequence of $K_{c}$ on concentration distribution vs ξ.

**Fig. 18:**
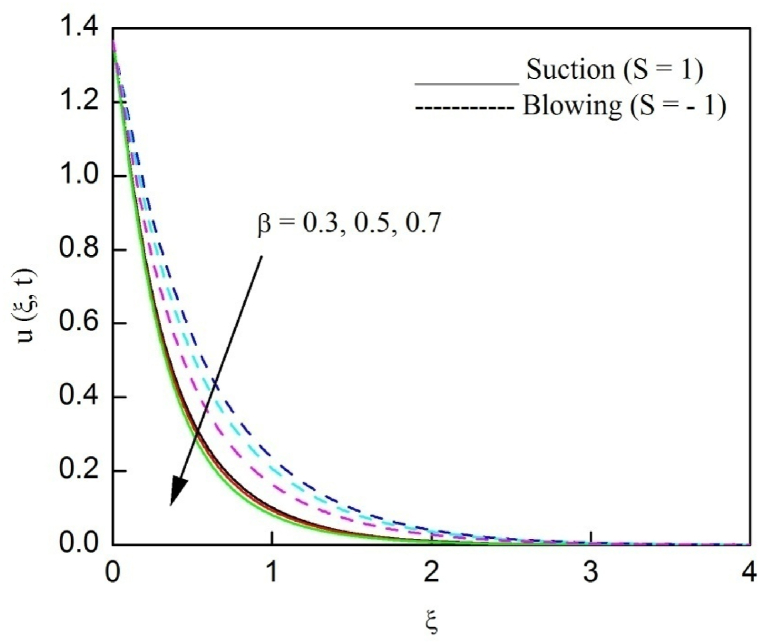
The consequence of β on velocity distribution vs ξ.

**Fig. 19:**
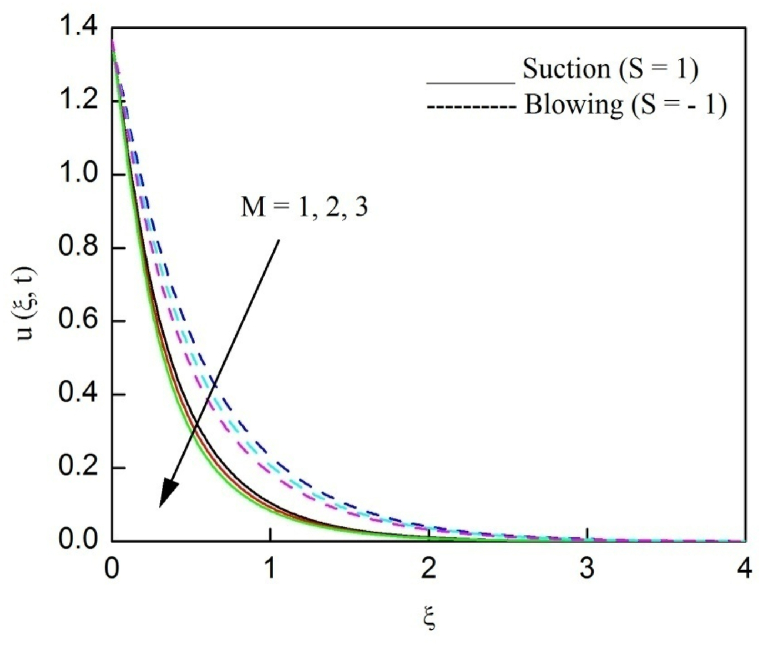
The consequence of M on velocity distribution vs ξ.

**Fig. 20:**
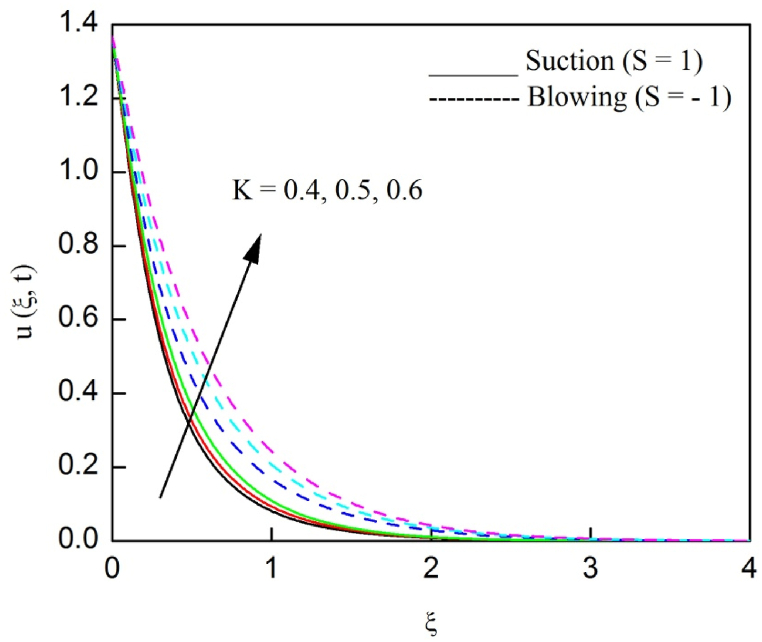
The consequence of K on velocity distribution vs ξ.

**Fig. 21:**
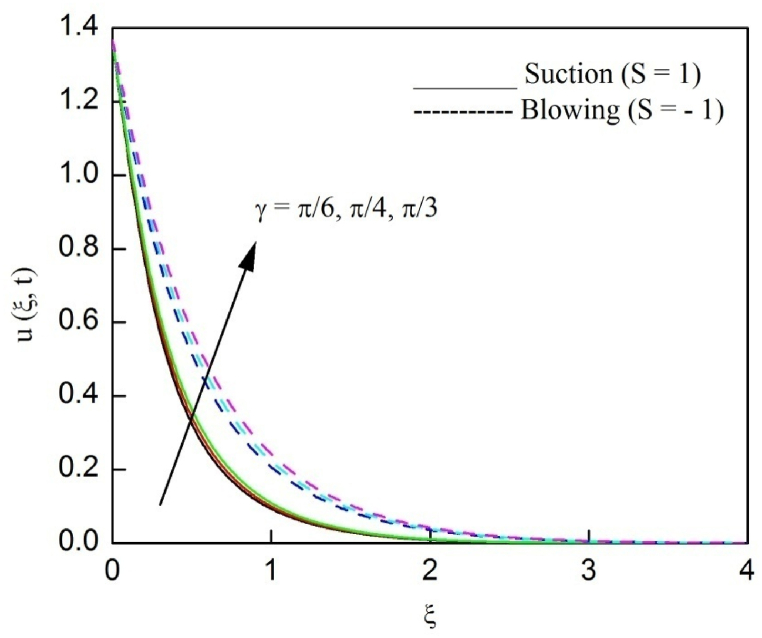
The consequence of γ on velocity distribution vs ξ.

**Fig. 22:**
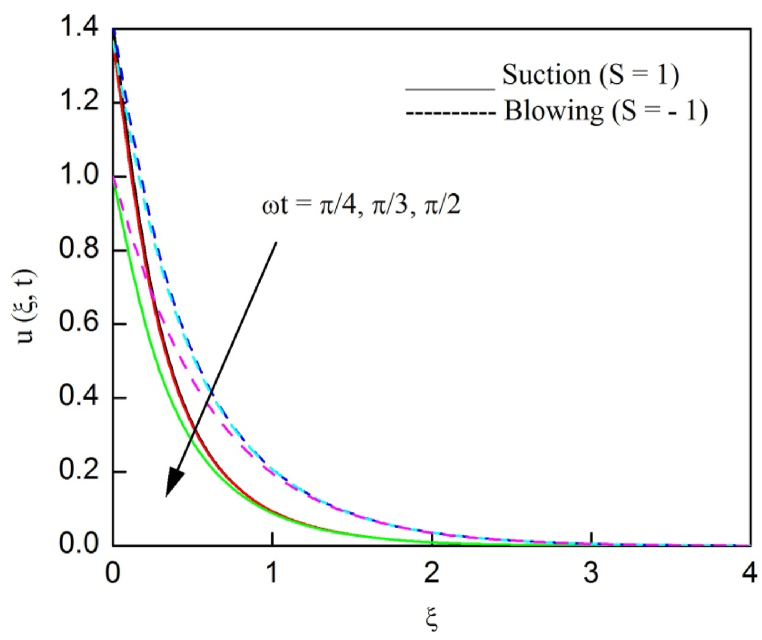
The consequence of ωt on velocity distribution vs ξ.

**Fig. 23:**
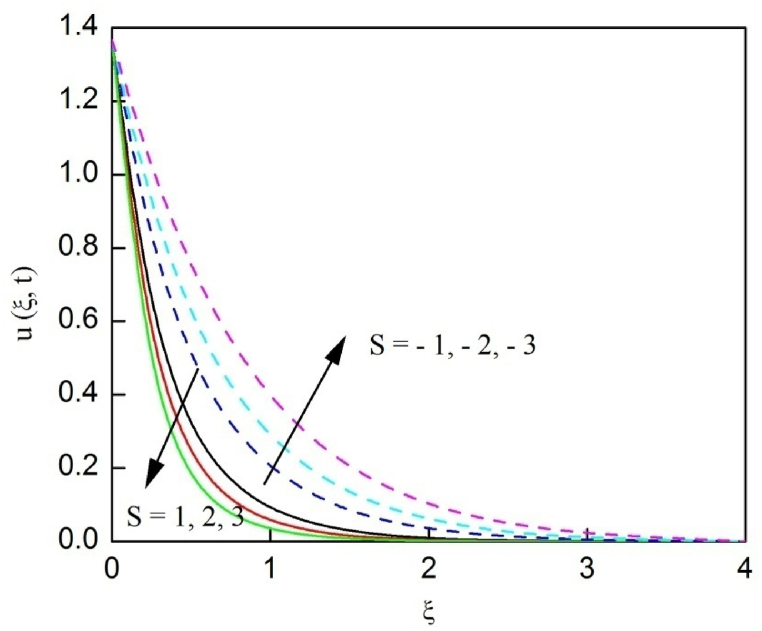
The consequence of S on velocity distribution vs ξ.

**Fig. 24:**
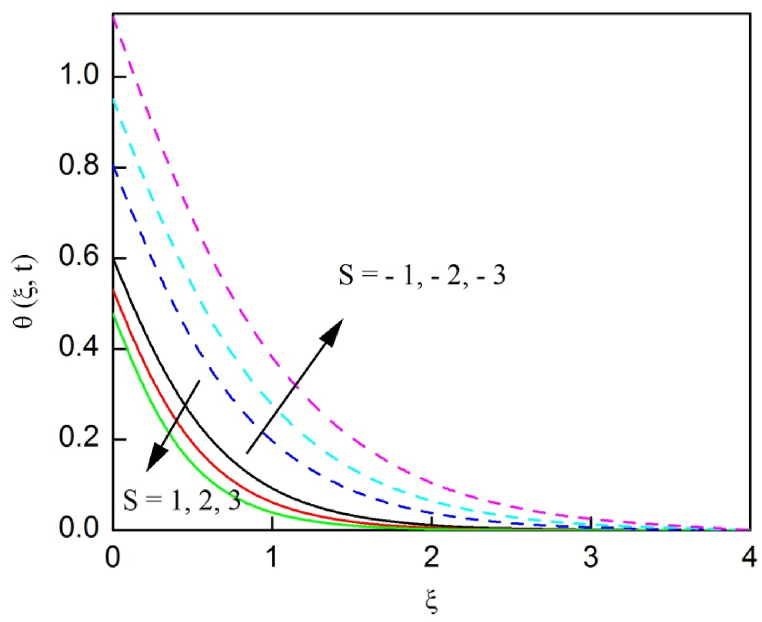
The consequence of S on temperature distribution vs ξ.

**Fig. 25:**
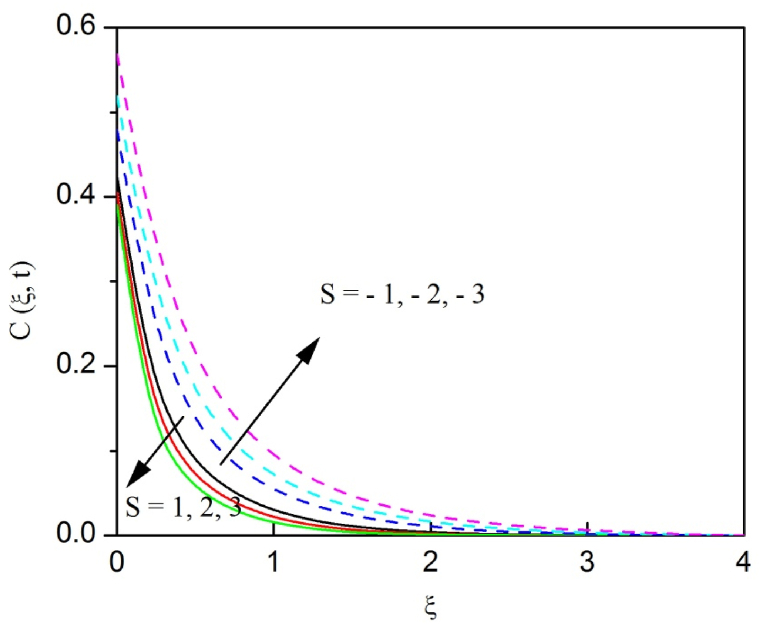
The consequences of S on concentration distribution vs ξ.

The profiles of velocity and temperature due to the dissimilarity of radiation parameter $N_{r}$ are described respectively, in Fig. 2, Fig. 3. It is clear that rising $N_{r}$ assist to broaden the velocity and temperature distributions. Physically, increasing $N_{r}$ values is the source to enlarge temperature boundary layer results in an enrichment of fluid temperature, consequently raise the fluid momentum. Fig. 4, Fig. 5 plotted to scrutinize the transmutation of the velocity and temperature owing to the Dufour number $D_{u}\text{,}$ respectively. It is remarked from these plots that, as we enlarge the Dufour number, the fluid velocity and temperature goes up. Physically, the Dufour number $D_{u}$ in the energy (heat) conserving equation indicates that the heat flux arising by a concentration gradient. As we increase $D_{u}$ values boosts transmission of heat in the boundary layer through the concentration gradient results into maximize the thermal boundary layer thickness consequently upsurge the fluid velocity. The alterations of the profiles of velocity and temperature for compounding heat absorption parameter $\varphi_{1}$ values are engraved in Fig. 6, Fig. 7, respectively. One can see that the velocity and temperature both decreases by increasing heat absorption parameter. This is fact in virtue of the bigger values of $\varphi_{1}$ lessen the temperature of the plate surface indebted to reduce fluid temperature eventually downturn is noted in the fluid velocity. Fig. 8, Fig. 9, respectively exhibits the graphical explanation of the effect of conjugate heat transfer parameter $\gamma_{1}$ on the profiles of velocity and temperature. From these figures, we observe that both fluid momentum and temperature enlarges with growing values of $\gamma_{1}\text{.}$ This fact occurs when increasing values of $\gamma_{1}$ implies higher heat transfer rate between the fluid and plate turn into enhance the fluid temperature, consequently hasten the fluid motion. The consequence of the radiation absorption parameter $\varphi_{2}$ on the profiles of velocity and temperature is analyzed respectively, in Fig. 10, Fig. 11, and increasing effect is noticed in the profiles of the velocity and temperature. The variation of both the velocity and concentration against the alteration of conjugate mass transfer $\gamma_{2}$ has been illustrated, respectively in Fig. 12, Fig. 13. These figures signify that increased values of $\gamma_{2}$ leads to significantly intensify the fluid velocity and concentration. This is because of larger values of $\gamma_{2}\text{,}$ there is a greater rate of mass transfer between adjacent layers convert into raise the concentration species ultimately speed up fluid flow. Fig. 14, Fig. 15 respectively, display the variance of the fluid velocity and concentration due to disparity in the Soret parameter $S_{r}\text{.}$ We can see that there is an upsurge in both flow velocity and concentration with increment in the values of $S_{r}\text{.}$ Physically, the (Soret) thermo-diffusion impact stimulate diffusion of solute in the boundary layer through thermal gradient. Therefore, enlarging $S_{r}$ values improve notably the concentration boundary layer thickness results marked improvement in the fluid concentration, ultimately heighten the fluid velocity. The fallout of chemical reaction parameter $K_{c}$ on the profiles of the velocity and species concentration is engraved in Fig. 16, Fig. 17, respectively. It is captured from these graphs that there is a down trend in both velocity and concentration profiles. Physically, snowballing values of $K_{c}\text{,}$ causes more species demolished through the chemical reaction results in a decrement of concentration boundary layer, eventually deplete momentum boundary layer thickness. Fig. 18 establishes the fluctuation of the profiles of velocity with heightening the Casson parameter β. It is perceived that there is a diminution in the fluid velocity as the values of β escalate. This is attributed to the reality that bigger values of β implies that the plasticity of the fluid enlarge, finally flow with diminutive velocity. The demesne of magnetic field strength M on the profiles of the velocity is observed in Fig. 19. This graph demonstrates that the fluid flowing with a small velocity by accumulating values of M this outcome is physically acceptable. Because of the enlarging values of M outgrowth a Lorenz force that works adverse to the flow path, in due time we see a deterioration in the fluid flow. The deviation of the profiles of velocity is ascertained in Fig. 20 for diverge permeability parameter K values. We can see from this plot that flow velocity is heighten with swelling values of K due to the diminution in the refusal of the porous medium. Fig. 21 describes the variation of the velocity profiles for dissimilar angled magnetic field γ This depiction exposed that; as we increase in the angled magnetic field γ values, accelerate the flow momentum. This signifies that when imposed magnetic force works on the back of the direction of the flow, then the magnitude of the Lorentz’s force become paramount, convincingly get better fluid motion. The pattern of the velocity profiles in contrast to the phase angle ωt for the distinct angles is illustrated in Fig. 22. This graph indicate that the velocity profiles lowering for increasing ωt Fig. 23, Fig. 24, Fig. 25 are depicted to review the profiles of velocity, temperature and concentration due to the alteration in the suction/blowing parameter. We observe from these graphs that the functioning of suction scale down the size of velocity, energy and concentration boundary layer related to the lowering profiles of momentum, temperature and concentration whereas contrary nature is identified for blowing. Because effective blowing always diminishes the wall shear stress and friction drag.

### 5.1 Skin-friction, Nusselt and Sherwood number

The numerical variance in the skin-friction (τ), Nusselt number $\left(N_{u}\right)$ and the Sherwood number $\left(S_{h}\right)$ on account of variation in the flow regulating parameters are showcased, respectively in Table 3, Table 4, Table 5. We observe from Table 3 that in the instances of suction and blowing, both the skin friction and Nusselt number heighten with advancing $\varphi_{1}$ and $\gamma_{1}$ values whereas there is a downturn is noted with increasing values of $N_{r},D_{u},\varphi_{2}$ and t. Table 4 exposed that the skin friction and Sherwood number escalates by strengthening values of $K_{c}$ and $\gamma_{2}$ whereas deterioration is acclaimed in both skin friction and Sherwood number because of higher $S_{r}$ values for both suction and blowing. We noted from Table 5 that for both actions of suction and blowing, the skin friction rises with enlargement of β and M values whereas a reversal trend is noted with K,γ, and ωt values.

**Table 3:** Numerical computation of τ and $N_{u}$.

$N_{r}$	$D_{u}$	$\varphi_{1}$	$\gamma_{1}$	$\varphi_{2}$	t	Nusselt number ($N_{u}$)		Skin friction (τ)	
						Suction	Blowing	Suction	Blowing
2	0.5	3	0.5	1	0.5	1.003890	0.952996	3.859158	4.191651
3	0.5	3	0.5	1	0.5	0.960982	0.886865	3.683712	3.873519
2	0.7	3	0.5	1	0.5	0.903870	0.911867	3.771378	4.059261
2	0.5	4	0.5	1	0.5	1.201208	0.984515	3.962226	4.443930
2	0.5	3	0.6	1	0.5	1.407576	1.257315	4.595818	4.642894
2	0.5	3	0.5	2	0.5	0.955938	0.932151	3.663168	3.740247
2	0.5	3	0.5	1	0.6	0.917408	0.835422	3.186366	4.054095

**Table 4:** Numerical computation of τ and $S_{h}$.

$S_{r}$	$K_{c}$	$\gamma_{2}$	t	Sherwood number ($S_{h}$)		Skin friction (τ)	
				Suction	Blowing	Suction	Blowing
2	4	0.5	0.5	1.363794	1.244562	3.859158	4.191651
3	4	0.5	0.5	1.276604	1.077904	3.748320	4.026015
2	5	0.5	0.5	1.411770	1.283934	4.298548	4.361193
2	4	0.6	0.5	1.818390	1.656534	3.771846	3.959307
2	4	0.5	0.6	1.267160	1.049884	3.186366	4.054095

**Table 5:** Numerical computation of τ.

β	M	K	γ (degree)	ωt (degree)	Skin friction (τ)	
					(Suction)	(Blowing)
0.5	2	0.5	30	60	3.859158	4.191651
0.7	2	0.5	30	60	3.958980	4.767084
0.5	3	0.5	30	60	4.046046	4.525335
0.5	2	0.6	30	60	3.285141	3.695625
0.5	2	0.5	45	60	3.724728	3.951513
0.5	2	0.5	30	90	2.546916	2.494971

## 6 Conclusion

In the current study, the inclined magnetic field, thermal radiation, suction/blowing and Soret-Dufour aspects on chemically reactive unsteady mixed convection Casson heat absorbing hydro-magnetic oscillatory flow in a flat semi-infinite vertical porous plate considering conjugate heating conditions at the boundary is numerically analyzed. The most important findings of the analysis follow:

(i) The velocity profiles speed up with increased $N_{r},D_{u},\varphi_{2},S_{r},K$ and γ values whereas there is a drop in the velocity profiles with enlarged $\varphi_{1},K_{c},\beta \text{,}$ and M values for suction and blowing cases. At the same time skin friction behaves contrary to the velocity.
(ii) The fluid velocity and skin-friction both enhanced with increasing $\gamma_{1}$ and $\gamma_{2}$, whereas we noticed a downturn with increasing ωt.
(iii) The fluid temperature is heightened while increasing $N_{r},D_{u}\text{,}$ and $\varphi_{2}$ whereas the Nusselt number act upon reversely.
(iv) An increase in $\varphi_{1}$ entails decrease of fluid temperature whereas Nusselt number shown opposite tendency.
(v) The fluid temperature and Nusselt number both advance on the rise $\gamma_{1}$.
(vi) The species concentration drained with rising $K_{c}$ whereas reverse behavior is noticed with surging $S_{r}\text{.}$ At the same time Sherwood number act upon oppositely.
(vii) The species concentration and Sherwood number both strengthen with increased $\gamma_{2}$.

## Data availability

The data used to backing the findings of the study are available within the article.

## CRediT authorship contribution statement

**B. Prabhakar Reddy:** Writing - review & editing, Writing - original draft, Validation, Software, Methodology, Investigation, Formal analysis, Data curation, Conceptualization. **Alijen Felician:** Writing - review & editing, Writing - original draft, Validation, Software, Methodology, Investigation, Conceptualization. **P.M. Matao:** Visualization, Supervision, Software, Methodology, Investigation, Formal analysis, Data curation, Conceptualization.

## Declaration of competing interest

The authors declare the following financial interests/personal relationships which may be considered as potential competing interests:B. Prabhakar Reddy reports administrative support was provided by The University of Dodoma. B. Prabhakar Reddy reports a relationship with The University of Dodoma that includes: employment. The authors declare that they have no known competing financial interests or personal relationships that could have appeared to influence the work reported in this paper. If there are other authors, they declare that they have no known competing financial interests or personal relationships that could have appeared to influence the work reported in this paper.
